# BudFinder: A Masked Auto-Encoder vision transformer framework for yeast budding detection and lifespan quantification

**DOI:** 10.1371/journal.pcbi.1013700

**Published:** 2026-05-18

**Authors:** Phuc Nguyen, Zahra Mousavi Karimi, Adrian Layer, Markus B. Wan, Hetian Su, Jeff Hasty, Nan Hao

**Affiliations:** 1 Department of Molecular Biology, School of Biological Sciences, University of California, San Diego, La Jolla, California, United States of America; 2 Department of Bioengineering, University of California, San Diego, La Jolla, California, United States of America; 3 Synthetic Biology Institute, University of California, San Diego, La Jolla, California, United States of America; University of Toronto Mississauga, CANADA

## Abstract

Studying replicative aging in yeast is a central component of aging research. Recent advances in time-lapse microscopy and microfluidics now enable continuous, high-resolution tracking of individual yeast cells throughout their lifespan. However, quantifying replicative lifespan from microscopy data remains labor-intensive, as it traditionally requires manual counting of cell division events for each cell. Recent deep learning-based approaches have begun to address this challenge by automating lifespan quantification. Here, we present a versatile image analysis framework that accurately detects yeast cell division events during replicative aging. To reduce the need for large, manually annotated datasets, we pretrain a Masked Autoencoder on large-scale (~250K), unlabeled yeast cell image crops. This self-supervised pretraining substantially lowers the amount of annotated data required to train a transformer model for division event detection. Moreover, our model is trained to directly identify budding events, eliminating dependence on arbitrary heuristics such as changes in cell area. By leveraging self-supervised learning, our approach only requires training data with fewer than 50 mother cells (~1,000 division events, which is significantly lower than reported in previous methods), while maintaining high detection accuracy.

## Author summary

Our work addresses a longstanding challenge in live-cell time-lapse microscopy analysis: automating cellular division tracking while minimizing the amount of training data required. Traditionally, scientists identify each division event by manually inspecting thousands of time-lapse images, a process that is both tedious and prone to bias. While automated tools exist, they often require large amounts of annotated data to work effectively. To overcome these barriers, we developed BudFinder, which can recognize and track cell divisions with far less training data. Using yeast replicative aging data as an example, we first trained a model to understand what a yeast cell “looks like”, using roughly 250K of raw yeast mother cell crops entrapped in our custom-built microfluidic device. Then, we taught it to detect budding events directly from time-lapse movies. This approach substantially reduces the need for manual labeling while maintaining high detection accuracy. By making high-throughput analysis of cellular division more accessible, our work paves the way for faster and more scalable quantification of cellular dynamics.

## Introduction

Recent advances in live-cell time-lapse microscopy have enabled researchers to continuously monitor the dynamic behaviors and molecular states of single cells over long timescales. Although these techniques reveal rich temporal phenotypes, analyzing such large image datasets remains a major challenge, as it requires segmentation, tracking, and quantification of individual cells across thousands of frames.

For example, the budding yeast *Saccharomyces cerevisiae* serves as a model for studying replicative aging in mitotically active cell types such as stem cells [1,2]. The total number of daughter cells each mother cell produces before ceasing to divide is defined as its replicative lifespan [3–5]. By following single mother cells over time, researchers can link molecular dynamics to lifespan outcomes.

**Fig 1:**
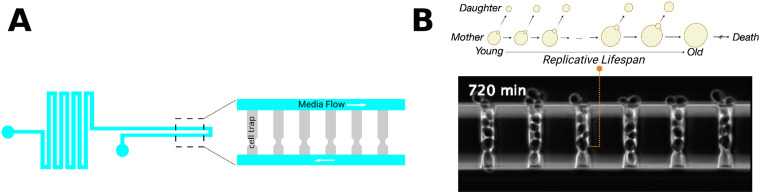
Overview of microfluidic platform. **(A)** Design layout of the microfluidic device. An inlet channel distributes fresh medium to cell traps, which immobilize single mother cells; dashed box highlights the trapping region. **(B)** Representative phase-contrast frame showing yeast mothers trapped at the bottom ends of individual traps while daughters are flushed away by the medium flow. Each mother cell is continuously tracked throughout its replicative lifespan.

Capturing these trajectories requires imaging individual cells every few minutes for several days to record each budding event and monitor fluorescent reporters of gene expression [6–12]. This is achieved by integrating microfluidic platforms (Fig 1A), which trap hundreds of mother cells in parallel side channels, with high-resolution time-lapse microscopy [13–20] (Fig 1B). This setup keeps each mother cell stably positioned in the imaging field for its entire lifespan while continuously flushing away daughter cells, resulting in detailed time-lapse movies that capture the full course of replicative aging.

However, the data volume is enormous: a single 100-hour microscopy run and a 15-minute sampling interval yields approximately 400 images per cell. Even with a lower limit of 100 mother-cell traps, a single experiment can yield thousands of raw images which must be managed before any preprocessing or quality-control steps can begin.

Traditionally, analysts comb through these stacks frame-by-frame, hand-annotating the exact moment of every bud emergence so that lifespan curves and cell-cycle metrics can be reconstructed [21]. At roughly 20–30 buds per cell, this translates to tens of thousands of manual annotations per dataset, which is an effort that is both time-consuming and prone to subjective drift. In short, while time-lapse microscopy coupled with microfluidics unlocks unprecedented single-cell resolution, the manual curation that it demands throttles high-throughput investigation into aging mechanisms.

Emerging deep-learning tools provide a promising avenue to break this bottleneck by automatically detecting cell division events - defined here as when a new bud is visibly formed - directly from raw image sequences, and opening the door to much larger, statistically powerful inferences. Recent studies demonstrate that modern deep-learning architectures can match human annotators in identifying budding events while operating far more efficiently [10,22]. For instance, YeastMate builds on Mask R-CNN, adding a custom head that simultaneously segments yeast cells and classifies lifecycle transitions [23]. Similarly, DetecDiv leverages the mother-machine platform by coupling a U-Net encoder with a temporal attention module to classify each frame into six distinct states: unbudded, small budded, large budded, dead, empty trap, or clogged trap. This frame-by-frame classification is then used to reconstruct replicative lifespan trajectories [24]. However, these methods report large amounts of annotated division data or complex classification schemes which are costly and labor intensive to generate. DetecDiv relied on approximately 200,000 brightfield images, with 1,000 frames manually labeled across six categories; YeastMate required roughly 17,000 annotated single cells, including roughly 2,900 budding events. This motivates the question of whether accurate division detection can be achieved with substantially less annotated data.

In contributing to this effort, we developed BudFinder, a Transformer-based framework optimized for efficient and accurate detection of cell division events in time-lapse microscopy data. Notably, BudFinder reduces the classification space to only two biologically meaningful states, budded vs. non-budded, which minimizes annotation complexity. Moreover, BudFinder achieves this performance with a dramatically smaller training dataset than previous methods: fewer than 50 mother cells (~1,000 annotated divisions) were sufficient for training. While prior studies do not explicitly state that large annotation volumes are required, there is currently no evidence that their performance would remain comparable under similarly limited training data. Accordingly, to our knowledge, BudFinder represents the first division-detection framework trained using substantially less manually annotated data than comparable methods such as DetecDiv and YeastMate.

**Fig 2:**
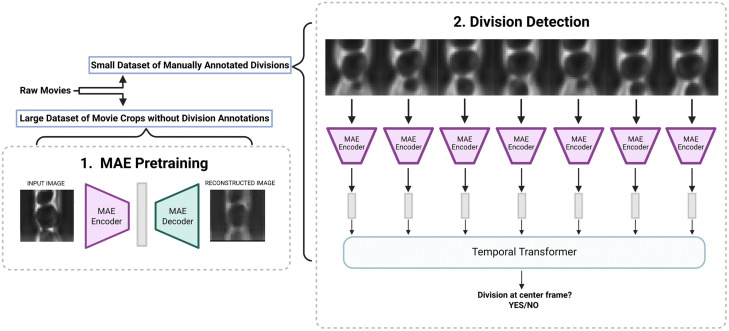
BudFinder framework for automated division detection. Raw time-lapse movies are used in two distinct training stages: [1] Masked Auto-Encoder (MAE) pretraining: single-frame crops from movies are used to pretrain an MAE in a self-supervised manner, learning visual representations without the need for division annotation data. [2] Division detection: temporally ordered sequences of MAE embeddings are passed to a lightweight temporal Transformer trained with annotated division events to predict whether a budding event occurs.

To achieve division detection with minimal annotated data, we hypothesized that the substantial demand for training data arises because the model must first learn to recognize and interpret the concept of a “cell” within an image before it can be effectively trained to detect complex cellular events such as division. Thus, we propose a two-stage framework as shown in Fig 2. In the first stage, a neural network can first be trained to recognize cellular imagery independently of the division-detection task. Concretely, we employ a self-supervised machine learning architecture which can be trained on large unlabeled image datasets to learn meaningful representations of objects within them. Based on the success of the Masked Auto-Encoder (MAE) architecture introduced in [25], we pretrain an MAE model on roughly 250K of images of yeast cell crops in the microfluidic device. The MAE encoder learns to produce compact “latent representations”, which contain local texture and global morphology features that are well suited to learning downstream tasks such as bud‑event detection. In the second stage, as shown in Fig 2 part 2, the pretrained MAE encoder is applied to each frame in a sequence of frames. A lightweight temporal Transformer head processes this sequence of embeddings instead of raw images, and is thus fine-tuned on less than 1,000 division-annotated clips to identify when division events occur [26]. Overall, our experiments demonstrate that the proposed two stage framework cuts annotation effort, lowering the barrier to high-throughput yeast replicative lifespan studies.

## Results

### BudFinder leverages MAE pretraining in a two-stage architecture for division detection

**Fig 3:**
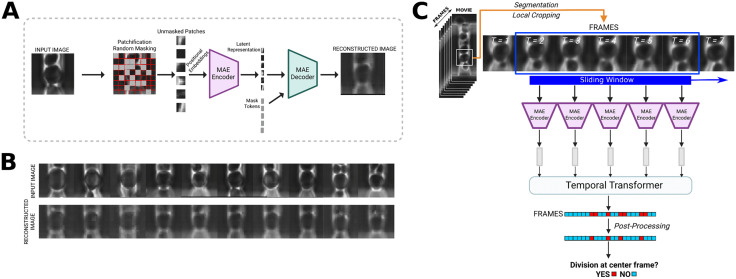
Learning single cell image features in MAE via reconstruction for integrating with temporal transformer for division detection. **(A)** Input image reconstruction process. Images are patchified and randomly masked, then positional embeddings are added and patches are encoded. Following encoding, mask tokens are introduced and the latent along with the mask tokens are decoded. Decoded patches are unshuffled using stored indices and the original image is then reconstructed. **(B)** Example of reconstructions produced by the pretrained MAE. Despite heavy masking during training, the MAE accurately reconstructs key cell morphology, indicating successful learning of yeast cell shape and internal structure. **(C)** After the reconstruction portion of training, weights are carried over, and the encoder is used on a frame-by-frame basis for each frame in an 11-frame tiff stack input. A temporal transformer takes the 11 latent representations as input and performs classification.

In this section, we describe the two stages of BudFinder in more detail (Fig 3). Starting from phase-contrast movies acquired in a microfluidic device, individual mother cells were segmented and tracked over time to generate aligned single-cell image sequences. Each frame was cropped around the tracked mother cell and processed to standardize cell position and scale. These single-cell crops served as input to the first stage:

#### Self-supervised MAE.

This stage was pre-trained on tens of thousands of yeast cell images to learn morphology-aware visual representations that can aid the second stage in division detection. As shown in Fig 3A, an MAE consists of an encoder and a decoder. During training, each input image was partitioned into patches, and only a randomly selected subset was fed to the encoder, which produces compact latent representations. The decoder uses these embeddings to infer the missing visual information. The encoder thus learns to produce latent representations that capture salient single-cell structure, as evidenced by accurate reconstruction of unobserved regions. After pretraining, the decoder is discarded while the MAE encoder is retained for the next stage. A more detailed overview of the MAE architecture is provided in the Methods section.

#### Division detection stage.

For division detection, temporally ordered stacks of consecutive frames were embedded using the pretrained encoder and analyzed by a lightweight temporal Transformer that integrated morphological information across time. Instead of performing multi-class lifecycle classification, BudFinder directly predicted whether a budding event occurred at the center frame of each temporal window. Frame-level predictions were post-processed to collapse contiguous positive predictions into single division events, yielding complete budding timelines for individual mother cells (Fig 3C). Summation of detected events produced replicative lifespan measurements, while summation of event timing enabled reconstruction of cell-cycle dynamics. Together, this end-to-end pipeline transformed raw time-lapse movies into quantitative aging metrics with minimal manual annotation.

### BudFinder framework accurately captures budding events and important aging-related metrics in time-lapse movies

**Fig 4:**
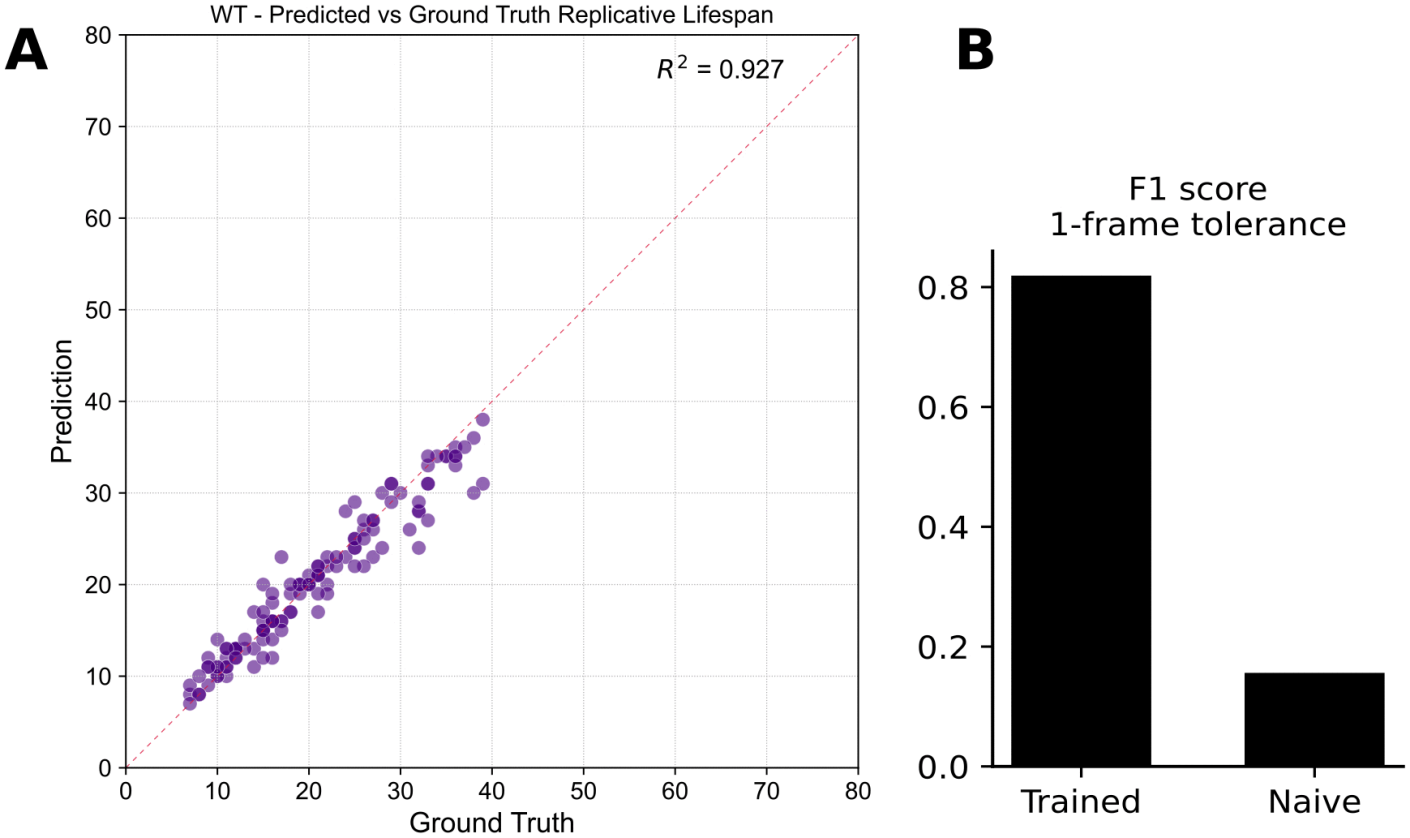
Automated division-detection model accurately reproduces ground-truth replicative lifespan and outperforms an untrained baseline. **(A)** Scatter plot of predicted versus manually annotated RLS for wild-type mother cells (n = 121). Points cluster tightly around the unity line (dashed red), indicating high agreement between model predictions and ground truth. **(B)** Bar graph comparing the division-event detection performance of the trained transformer model to a naïve heuristic using a ± 1-frame tolerance. Trained-versus-untrained comparisons are presented only as diagnostic controls, confirming that model accuracy depends on learned visual and temporal representations rather than intrinsic architectural priors.

To assess the accuracy of our automated cell division detection pipeline, we compared the predicted replicative lifespan (RLS) against manually annotated ground truth data across wild-type yeast cells. Importantly, all training and evaluation datasets were derived from independent experimental replicates collected on different imaging days, and no training data were used during validation or testing. The predicted RLS closely matched the ground truth values, with a high coefficient of determination (R² = 0.927), indicating strong concordance between model predictions and human annotations (Fig 4A). We further evaluated the performance of the division detection model using F1 score with a 1-frame tolerance window. The trained model significantly outperformed an untrained model baseline, achieving an F1 score of approximately 0.8 compared to <0.1 for the naïve approach (Fig 4B). Additionally, the model achieved a recall of 0.81 and a precision of 0.83, indicating that the majority of true division events were correctly detected. In terms of RLS prediction error, the standard deviation from ground truth was under 3.5 divisions, demonstrating the model’s ability to detect divisions with high temporal precision despite the spatial and temporal complexities observed in the timelapse imaging dataset.

Given our framework’s ability to reliably detect budding events, we next asked whether its performance remained robust across the morphological differences characteristic of the two canonical yeast aging trajectories. In previous work, we stratified the wild-type population into two distinct phenotypes, referred to as Mode 1 and Mode 2 aging [11].

**Fig 5:**
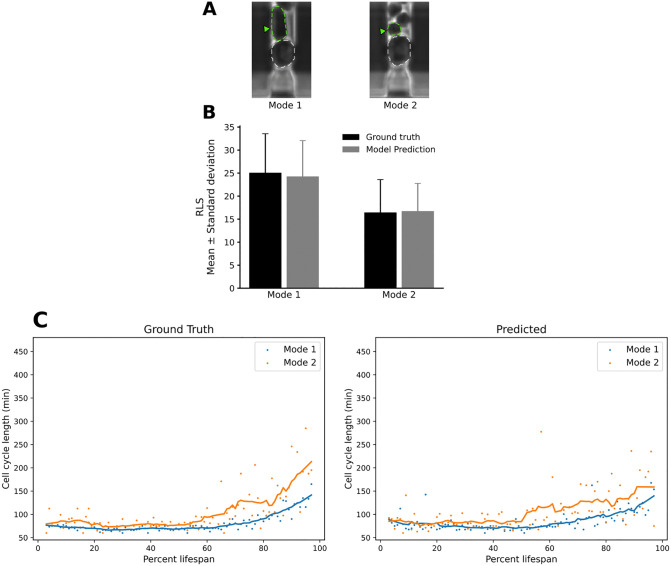
Automated division-detection accurately reproduces budding events irrespective of aging mode and daughter cell morphology. **(A)** Representative phase-contrast images from the mother-machine device illustrating the two budding patterns observed in wild-type cells. Mode 1 shows elongated daughter cells (left), whereas Mode 2 cells exhibit small round daughter cells (right). Green arrowheads mark daughter cells; white dashed outlines delineate the mother cell boundary. **(B)** Mean ± SD replicative lifespan for Mode 1 and Mode 2 mothers measured by manual annotation (black bars) versus BudFinder model (grey bars). Model predictions closely match ground truth scores for both modes (two-tailed paired t-test, n.s. for Mode 1 and Mode 2). **(C)** Cell-cycle length trajectories over the course of life, expressed as a percentage of each cell’s total lifespan. Scatter points represent individual cycles; solid lines are Savitzky–Golay–smoothed trajectories (left, manual ground-truth annotations; right, model predictions). The model recapitulates the rapid budding characteristic of young cells, followed by progressive late-life cell-cycle lengthening, with Mode 2 cycles consistently longer than Mode 1 across both ground-truth and BudFinder model outputs.

Mode 1 cells, roughly half of the cohort, switch from producing small round daughters to generating elongated daughters late in life and exhibit pronounced nucleolar enlargement and fragmentation, signaling nucleolar decline (Fig 5A, Top Left). Mode 2 cells, in contrast, continue budding small round daughters until death; their nucleoli remain morphologically unaltered, but they display progressive mitochondrial aggregation, a hallmark of mitochondrial dysfunction (Fig 5A, Top Right). Using these criteria, we labeled each cell track as Mode 1 or Mode 2 and evaluated the division-detection model accordingly. This partitioning allowed us to determine whether the model can recover replicative lifespan and cell-cycle dynamics despite the divergent morphological outcomes that define each aging mode. The predicted mean RLS values were consistent with the corresponding ground truth distributions across both aging modes, with no significant differences observed. This suggests that the model generalizes well across divergent aging trajectories and daughter cell morphologies (Figs 5B, 5C, S1 and S2).

Furthermore, to evaluate model robustness under physiological perturbation, cells were treated with salicylic acid, a perturbation known to inhibit heme biosynthesis and mitochondrial function, thereby markedly slowing cell-cycle progression. As shown in S3 Fig, BudFinder accurately predicted the corresponding reduction in division events, in agreement with manual ground-truth annotations. These results demonstrate that the classifier does not generate spurious division predictions when budding-associated morphological transitions are suppressed, supporting both the specificity of the model and its robustness to altered growth conditions. Overall, these results demonstrate that the division detection pipeline accurately reconstructs replicative lifespan and cell cycle dynamics, achieving high accuracy annotation performance while enabling high-throughput analysis of aging phenotypes.

### BudFinder generalizes across divergent aging phenotypes while maintaining accuracy at both frame and lifespan scales

We next tested whether the model maintains its accuracy in obtaining replicative aging information on short-lived or long-lived mutants in yeast: short-lived *sir2*∆ and a long-lived synthetic oscillator developed by Zhou et al. [12]. We then assessed the performance between ground-truth data and the model at two complementary scales.

**Fig 6:**
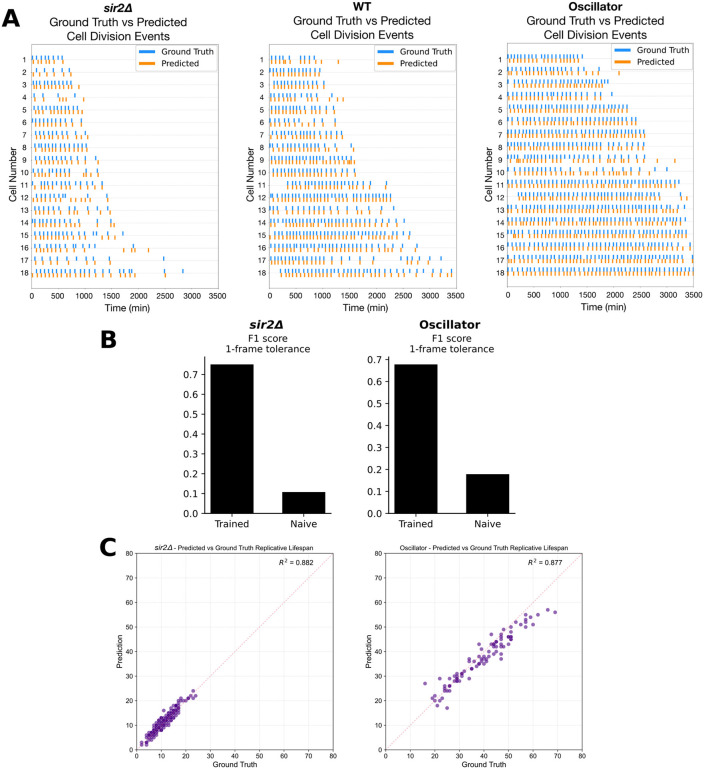
BudFinder reproduces manual budding timelines, detects divisions in genetically perturbed strains, and accurately predicts replicative lifespan across diverse genetic backgrounds. **(A)** Raster plots show ground-truth (blue) and model-predicted (orange) budding events for at least n = 15 representative mother cells per strain. The near-perfect superposition of orange and blue ticks across all three panels demonstrates that BudFinder preserves frame-level accuracy despite the distinct division frequencies and lifespan trajectories characteristic of each genotype, confirming its robustness to genetic perturbations. Prediction accuracy was quantified via the Hungarian algorithm. In the WT dataset, 54.5% of predictions matched ground truth exactly, with a mean error of +0.27 frames indicating a mild late bias; 30.3% of events were late, 8.1% early, and 7.1% unmatched. The sir2Δ dataset exhibited late bias, with 32.4% exact matches, 58.5% late predictions, 3.1% early, and 6.0% unmatched. The oscillator dataset showed a moderate early bias, with 40.8% exact matches, 35.5% early predictions, 15.6% late, and 8.1% unmatched. **(B)** Bar plots show F1 scores calculated with a 1-frame tolerance for the trained division-detection model (left bar in each panel) versus a naïve baseline (right bar) in sir2Δ cells (left) and an engineered long-lived oscillator strain (right). The trained model achieves F1 ≈ 0.75 in sir2Δ and F1 ≈ 0.68 in the oscillator, more than four-fold higher than the baseline in both cases. **(C)** Scatter plots compare predicted versus ground-truth replicative lifespan for sir2Δ (left, n = 172) and the engineered oscillator strain (right, n = 90). Each point represents one mother cell; the red dashed line denotes the 1:1 correspondence.

1. *Frame-level event detection:* For each strain, we compared the predicted and ground-truth budding timelines. Raster plots for 30 representative *sir2*Δ mother cells show that the model’s predicted division ticks (orange) align almost perfectly with the ground-truth ticks (blue) (Fig 6A). Across 1,919 annotated frames, the model correctly identified 89.6% of predictions occurring within ±2 frames of the manual labels, corresponding to an F1 score of 0.75 under a 1-frame tolerance (Table 1 and Fig 6B, Left). Similarly, for the oscillator strain, the model captured 82.7% of 3,549 annotated budding events within ±1 frame, and 90.7% within ±2 frames of the manual calls, yielding an F1 score of 0.68 at the 1-frame tolerance (Table 1 and Fig 6B, Right). In the oscillator strain, BudFinder prioritized sensitivity, reaching a high recall of 0.85 with a moderate precision of 0.56. For *sir2*Δ, performance was more well balanced, with both recall and precision near 0.75, demonstrating consistent division detection despite altered lifespan regulation. Frame-level event detection therefore remains high despite both mutant’s distinct cell cycle dynamics.

**Table 1:** Frame-level agreement between BudFinder model and manually annotated budding events across three genetic backgrounds. Percentages indicate the fraction of predicted division ticks that coincide with the ground-truth annotation in the same frame (Exact match) or within a tolerance window of ±1 or ±2 frames. Even under the rapid cycling of the oscillator strain and the shortened lifespan of sir2Δ, the model captures > 78% of events within ±1 frame and > 89% within ±2 frames, demonstrating robust temporal precision comparable to wild-type performance.

Condition	Exact match	± 1 frame	± 2 frames
**WT**	54.9%	88.7%	91.8%
**Oscillator**	41.1%	82.7%	90.7%
***sir2Δ***	32.5%	78.4%	89.6%

1. *Replicative lifespan detection:* Summing the detected events yields a predicted lifespan for every mother cell. Scatter plots of predicted versus measured lifespans show tight agreement across both genotypes: R^2 = 0.882 for *sir2*∆ and 0.877 for the oscillator (Fig 6C). Notably, the regression slopes did not differ significantly from 1, demonstrating that the model generalizes without strain-specific recalibration. Together, these results confirm that our self-supervised temporal-Transformer pipeline reproduces both the fine-grained cell-cycle timing and the coarse-grained lifespan statistics required for high-throughput aging studies. In summary, although training was restricted to WT cells under favorable nutrient conditions, the model robustly generalizes across diverse strain backgrounds.

### Division counting and lifespan prediction perform robustly across imaging platforms

**Fig 7:**
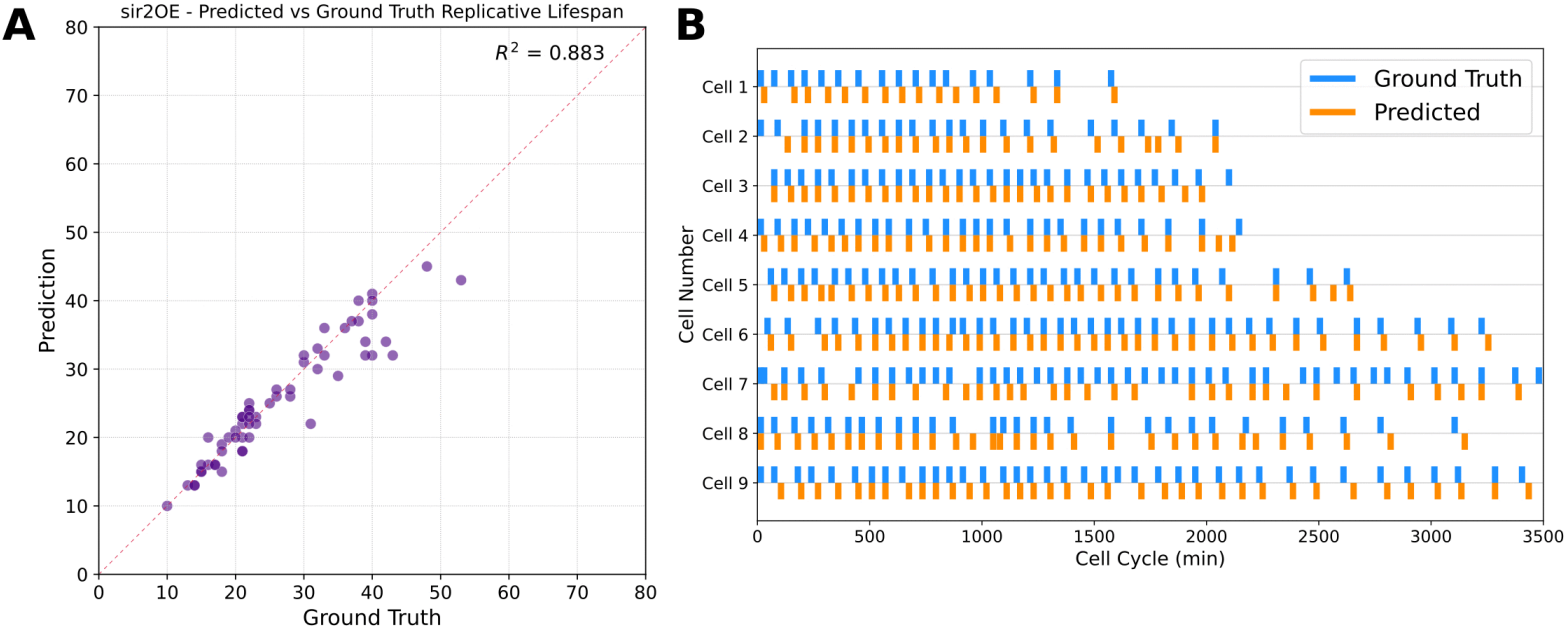
Benchmarking division detection performance across different imaging platforms. **(A)** Scatter plot comparing predicted versus ground-truth replicative lifespan for sir2 overexpression (n = 58). **(B)** Comparison of predicted and ground-truth division events for representative single cells.

To determine whether BudFinder’s performance is platform-independent, we evaluated the model on a dataset that was acquired using a distinct imaging platform under a different optical configuration and exposure settings that were not represented during training. Without retraining or fine-tuning, BudFinder accurately predicted replicative lifespan across these conditions, yielding strong agreement between predicted and ground-truth division counts (Fig 7A; R² = 0.883). Additionally, the division detection performance reaches an F1 score of 0.74 (recall = 0.79, precision = 0.69).

At the single-cell level, predicted division events closely recapitulated manually annotated budding timelines (Fig 7B). Together, these results demonstrate that BudFinder’s division detection and lifespan prediction are robust to variations in imaging hardware and acquisition conditions.

## Discussion

In this study, we present BudFinder, a Transformer-based framework that leverages MAE pretraining to enable accurate and efficient detection of yeast cell divisions. By combining self-supervised representation learning with a lightweight temporal Transformer, our approach reduces the annotated training data requirement, while maintaining state-of-the-art accuracy in reconstructing replicative lifespan trajectories. Importantly, BudFinder faithfully recapitulates budding timelines across wild-type and mutant strains. This demonstrates the model’s robustness to morphological variability, divergent aging modes, and altered cell cycle dynamics.

The novelty of our work lies in decoupling cell representation learning from division-event detection. We show that an MAE trained on raw yeast cell images provides morphology-aware embeddings that can be transferred to downstream classification tasks with minimal supervision. This strategy circumvents the need for vast, manually curated datasets, which is a persistent bottleneck in the field, and enables scalable, high-throughput analysis of time-lapse images.

Our results demonstrate that BudFinder exhibits a high degree of robustness across different imaging platforms and experimental configurations within our laboratory. In these settings, the model achieved strong division detection and replicative lifespan reconstruction performance without requiring retraining or fine-tuning, indicating that the learned representations capture broadly relevant morphological and temporal features rather than platform-specific artifacts (Fig 7). For deployment in other laboratories, we expect that light fine-tuning of the temporal prediction head using a modest amount of annotated data will often be sufficient to achieve good performance, particularly when imaging conditions are broadly comparable. Full retraining of the pipeline, especially the self-supervised pretraining stage, may yield optimal results in cases where optical settings differ substantially, as this allows the model to fully adapt to dataset-specific characteristics.

With respect to temporal resolution, BudFinder was designed for the acquisition regimes commonly used in multi-day yeast aging experiments. In our previous microfluidic yeast aging studies, a 15-minute imaging interval was empirically identified as optimal: it reliably captures budding-associated morphological transitions while minimizing phototoxicity and physiological perturbations that arise at higher sampling frequencies [6,8,10–12]. Increasing temporal resolution beyond this interval can lead to altered growth dynamics and inconsistent lifespan measurements. Because bud emergence and associated morphological changes occur on timescales well above 15 minutes, BudFinder does not rely on fine-grained temporal interpolation but instead detects robust morphological signatures of budding within a sliding temporal window. As long as these transitions are consistently captured within the input window, the model can reliably identify division events. However, in principle, denser sampling would introduce additional intermediate frames between division states, increasing temporal redundancy and potentially simplifying classification, and therefore would be helpful for projects that allow high-frequency imaging.

While BudFinder demonstrates strong generalization, certain limitations remain. Although our framework reliably identifies division events, it does not explicitly monitor daughter morphology, limiting its ability to quantify features such as bud volume growth. Future work may integrate instance segmentation or multimodal architectures to extend BudFinder’s capacity beyond division counting.

As with most deep learning–based classifiers, occasional false positive and false negative predictions occur. We speculate that false positives arise from transient cellular motions or shape changes that resemble the dynamic morphological patterns associated with budding, whereas false negatives may occur when bud emergence is unusually gradual or when buds are temporarily out of the focal plane, reducing visual salience. Importantly, these errors occur at low frequency and do not compromise overall performance or downstream biological conclusions.

The implications of BudFinder extend beyond yeast aging. Our framework highlights the utility of self-supervised pretraining in cellular image analysis, particularly for domains where annotated datasets are scarce. We envision that similar approaches could be applied to other single-cell imaging platforms, ranging from bacterial colony dynamics to mammalian stem cell differentiation. More broadly, the statistical efficiency of BudFinder opens the door to systematic, large-scale exploration of genetic and environmental factors that modulate replicative lifespan. By minimizing manual annotation and enabling large-scale, quantitative analysis, BudFinder provides a generalizable framework that advances live-cell imaging and deepens our understanding of dynamic cellular processes.

## Methods

1. *Experimental Setup*:

### Microfluidic device fabrication & setup

The microfluidic devices and experiments were conducted according to previously established protocols [6,8,12]. In summary, a microfluidic device was fabricated using a 4-inch silicon wafer patterned with SU-8 2000 series photoresists following standard photolithography techniques. PDMS devices were then cast by pouring a Sylgard 184 mixture onto the wafer molds, degassing under vacuum, and curing for at least 1 hour. The PDMS devices were then cured at 60C overnight. The PDMS replicas were cut, punched, cleaned, and irreversibly bonded to glass slides using oxygen plasma. Detailed protocols for microfluidic device fabrication can be found in (O’Laughlin et al., 2023) [13]. Once the devices were prepared, to perform the experiments, yeast cells were cultured in SC medium with 2% glucose until reaching an OD600 of 0.8. The microfluidic devices were vacuum-treated for 20 minutes, coated with 0.075% Tween-20, and then placed on a 30°C incubator-equipped inverted microscope. Media ports were connected to syringes with synthetic dextrose media containing 0.04% Tween-20, positioned to enable gravity-driven flow. Yeast cells were loaded into the device, followed by media tubing reconnection. Flow rates were maintained at ~2.5 mL/day with waste collected in tubes.

### Single-cell aging time-lapse microscopy

Time-lapse microscopy was performed using a Nikon Ti-E inverted fluorescence microscope equipped with an EMCCD camera (Andor iXon X3 DU897) and a Spectra X LED light source. Imaging was conducted with a CFI Plan Apochromat Lambda DM 60×oil immersion objective (NA 1.40, WD 0.13 mm). Phase images were captured every 15 minutes over a duration of 90 hours, with an exposure setting of 50 ms (Microscope 1). To assess the generality of the model, the evaluation dataset for Fig 7 was acquired using different instrument and acquisition settings, as follows: Imaging was performed on a Nikon Ti-E inverted fluorescence microscope of the same model as used during training, but on a distinct instrument (Microscope 2). Phase images were collected using an exposure time of 100 ms (in comparison to 50 ms), with variations in illumination conditions, including differences in lamp brightness and camera EM gain. These differences introduced additional variability in image intensity and contrast (S4 Fig) relative to both the training data and the evaluation datasets presented in Figs 4 and 6.

1. *Model Architecture:*

### Overview

Our model was trained in two phases. In the first phase, an MAE was trained to reconstruct single-cell yeast images, enabling the encoder to learn morphology-aware representations. In the second phase, the pretrained encoder along with a classification head was trained to detect budding events from a temporal sequence of images. A final post-processing module takes in frame-level classification of a movie and outputs the following: (i) total replicative lifespan for each mother cell, (ii) the timing of division events, and (iii) frame-wise probabilities of division across the entire lifespan.

2.1 **Data preprocessing**

Raw microscopy data were acquired as 90-hour multichannel nd2 recordings and converted to plane-wise TIFFs. Frames were aligned to correct stage drift, and segmentation masks for individual cells were generated using Cellpose [27]. Segmentation quality was manually inspected and found to be consistent with the performance reported by Stinger et al. [27]; Because segmentation is not the core focus of our methodology, any segmentation tool that provides sufficiently accurate masks for reliable cell localization would be suitable for downstream representation learning and event detection. The resulting segmentation masks were used to localize each cell and define a cropped region centered on the mother cell for downstream analysis. Accurate segmentation enables more precise centering within each crop, reducing positional variability and facilitating more consistent learning of cellular representations by the model. Frame-resolved positional and morphological information such as mother cell position, area, aspect ratio, and flat-field-corrected fluorescence intensities were then extracted with ictrack [28] and assembled into a phenotype table (CSV format) that stored mother cell position, area, aspect ratio, and flat-field-corrected fluorescence intensities. For each 15-minute frame, one row per mother cell was appended, yielding an analysis-ready dataset in less than 24h. This preprocessing workflow not only produced high-quality morphometric and fluorescence measurements suitable for immediate statistical analysis but also streamlined downstream representation learning and event classification.

2.2 **Training dataset for MAE pretraining and division detection**

We leveraged previously published long-term single-cell imaging data from Zhou et al. for both MAE pretraining and downstream division detection [12]. Concretely, the MAE model was trained on unlabeled image crops derived from all frames across all single-cell trajectories in the dataset, comprising approximately 2,167 mother cells representing multiple genotypes. Crops were generated by extracting centered 224 × 224 px regions for each mother cell at every frame, followed by resizing to 64 × 64 px. The resulting training and validation sets comprised approximately 250,000 and 64,000 frame-level samples, respectively.

Training of the cell division detection model was subsequently performed using a small subset of wild-type mother cell movies (approximately 70 mother cells) from the same dataset. Samples were constructed by generating temporal 224 × 224 px TIFF stacks, each comprising 11 consecutive image crops (the frame of interest together with five preceding and five subsequent frames). Cells were randomly partitioned into training (80%) and validation (20%) sets, and all frames from a given cell were assigned exclusively to one split.

2.3 **Masked Auto-Encoder pre-training**

Our implementation follows the architecture described in *Masked Autoencoders Are Scalable Vision Learners* [25]. The model comprises of an encoder, a decoder, and a learnable mask token. During training, each input image crop was partitioned into patches, and a substantial fraction of the patches were randomly masked. The remaining unmasked patches were passed through the encoder to produce latent representations of each patch. The decoder then received these latent representations together with mask tokens inserted at the positions of the missing patches. Using this combined input, the decoder attempted to reconstruct the original image. The model was trained end-to-end using the reconstruction loss on the image, computed using L2 norm. This reconstruction objective enabled the model to learn informative visual representations without requiring manual annotations. Because Vision Transformers do not inherently preserve spatial relationships, positional embeddings (learnable vectors that encode the spatial location of image patches) were added to all tokens to provide explicit spatial information [29].

Training incorporated multiple strategies designed to improve optimization stability, representation quality, and generalization. First, we employed an exponential moving average (EMA) model, in which a secondary set of model weights was updated as a running average of the primary network parameters throughout training. This approach reduces high-frequency parameter fluctuations and typically yields more stable and better-generalizing representations.

To further stabilize optimization, we used a composite learning-rate schedule consisting of an initial warm-up phase followed by cosine annealing with periodic restarts. The warm-up phase gradually increased the learning rate from a small value, mitigating early-training instabilities commonly observed in Transformer-based architectures. After warm-up, cosine annealing enabled smooth learning-rate decay, while periodic restarts allowed the optimizer to escape shallow local minima and explore alternative regions of the loss landscape.

Because MAE pretraining benefits from large effective batch sizes, gradient accumulation was used to simulate larger batches than permitted by hardware memory constraints. Gradients were accumulated across multiple forward-backward passes prior to each optimizer update, improving gradient estimates without increasing memory usage.

Model parameters were optimized using the Adam optimizer with weight decay. Adam’s adaptive moment estimation improves convergence behavior in high-dimensional parameter spaces, while weight decay regularization helps prevent overfitting and encourages smoother representations. Together, these techniques promote stable long-horizon training and improved feature learning.

The MAE model was trained for more than 500 epochs to ensure adequate convergence of the self-supervised objective. Model selection was performed by monitoring validation loss, and the checkpoint corresponding to the best validation performance was retained for downstream supervised division detection tasks.

2.4 **Cell division detection training**

Supervised division detection used temporal single-cell TIFF stacks of 11 consecutive crops. Each stack was then patchified, linearly projected into token embeddings, and augmented with positional embeddings, analogous to the preprocessing procedure used during MAE pre-training. To preserve the ordering of frames within each stack, sinusoidal temporal embeddings were added to encode the relative position of each frame in the sequence. This model leverages weights imported from the encoder portion of the MAE, but is modified for classification tasks.

The division detection model reuses the encoder weights learned during MAE pre-training but adapts the architecture for a supervised classification task. Specifically, the Vision Transformer encoder was retained, while the decoder used during self-supervised reconstruction was removed. In contrast to the pre-training stage, no image patches were masked, allowing the model to leverage the full visual information available in each frame.

Within each stack, frames were processed individually by the encoder. A classification token was prepended to the patch tokens for each frame, enabling the model to produce a compact summary representation capturing the visual features of that frame. The resulting frame-level embeddings were then passed to an additional Transformer module designed to integrate information across time. This temporal modeling stage allows the network to capture dynamic morphological changes associated with budding events, rather than relying solely on single-frame appearance. Each frame was mapped to a binary classification score (logit) representing the model’s confidence for budding versus non-budding. These scores were converted into probabilities using a softmax function, which normalizes the outputs into values between 0 and 1. Because budding events are inherently sparse relative to non-budding frames, this task exhibits class imbalance. To mitigate potential bias toward the majority class, we employed balanced batch sampling during training. Each mini-batch was constructed to contain equal numbers of budding and non-budding samples.

2.5 Post-processing

Predictions generated across the full lifespan of each cell were then aggregated. To avoid counting the same budding event multiple times, post-processing was applied such that consecutive positive predictions were merged into a single division event.

## Supporting information

S1 Fig Mean ± SD replicative lifespan for Mode 1 and Mode 2 mothers measured by manual annotation (black bars) versus BudFinder model (grey bars) for *sir2*∆. (TIF)

S2 Fig Cell-cycle length trajectories over the course of lifespan for *sir2*∆. (TIF)

S3 Fig BudFinder accurately detects division events following salicylic acid treatment, which perturbs heme biosynthesis and mitochondrial activity and consequently slows cell-cycle progression. Despite suppression of continuous budding, BudFinder correctly predicted the absence of division events, in agreement with ground-truth observation. This demonstrates that BudFinder maintains accurate division detection despite cell cycle progression alterations.
(TIF)

S4 Fig Phase comparison between imaging platforms. (Left) Distribution of phase values across all cells from Microscope 1 and Microscope 2. (Right) Mean phase values per cell for each microscope. ****p < 0.0001. Microscope 1 images were with a 50 ms exposure, whereas Microscope 2 images were acquired using a 100 ms exposure, with variations in lamp brightness and camera EM gain.
(TIFF)
