# Supplementary figures and images for: BudFinder: A Masked Auto-Encoder vision transformer framework for yeast budding detection and lifespan quantification
Source: PLoS Comput Biol. 2026 May 18;22(5):e1013700. doi: 10.1371/journal.pcbi.1013700 (PMC13193611; doi:10.1371/journal.pcbi.1013700)

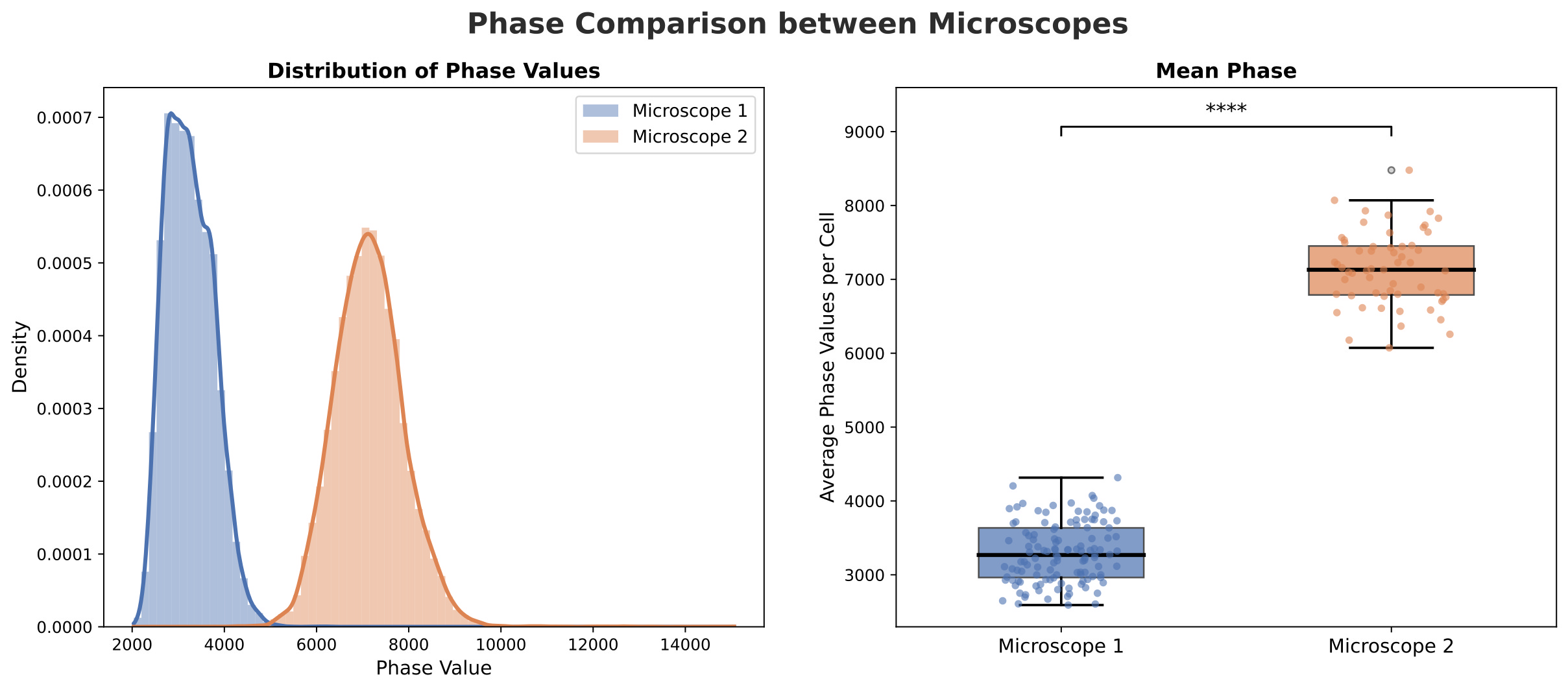

Supplement: S4 Fig — (Left) Distribution of phase values across all cells from Microscope 1 and Microscope 2. (Right) Mean phase values per cell for each microscope. ****p < 0.0001. Microscope 1 images were with a 50 ms exposure, whereas Microscope 2 images were acquired using a 100 ms exposure, with variations in lamp brightness and camera EM gain. (TIFF) [file pcbi.1013700.s004.tiff]
